# In-Person Visits Before Initiation of Telemedicine for Mental Illness

**DOI:** 10.1001/jamahealthforum.2024.0234

**Published:** 2024-04-05

**Authors:** Ateev Mehrotra, Alisa B. Busch, Lori Uscher-Pines, Pushpa Raja, Haiden A. Huskamp

**Affiliations:** 1 Department of Health Care Policy, Harvard Medical School, Boston, Massachusetts; 2 Beth Israel Deaconess Medical Center, Boston, Massachusetts; 3 McLean Hospital, Belmont, Massachusetts; 4 RAND Corporation, Arlington, Virginia; 5 Greater Los Angeles Veterans Affairs Medical Center, Los Angeles, California

## Abstract

This cross-sectional study examines how often patients had an in-person visit before initiating telemedicine for mental illness between 2019 and 2022.

## Introduction

Debate continues regarding whether in-person visits should be required for patients receiving telemedicine. In 2020, Congress permanently expanded Medicare telemental health coverage but required an in-person visit within 6 months before a first telemental health visit. Many organizations are concerned this in-person requirement will impede patient access to care.^1,2^ Subsequent legislation delayed the requirement until January 2025.^3,4^

Given little empirical research on this topic, we examined how often patients had an in-person visit before their first telemental health visit between 2019 and 2022 (when in-person requirements were not in place). The results may shed light on whether clinicians currently view in-person visits as clinically necessary and how clinical practice may need to change to comply with new rules.

## Methods

The Harvard University Institutional Review Board approved this cross-sectional study and waived informed consent because deidentified data were used. The STROBE reporting guideline was followed.

Using 2018-2022 national data for 100% of traditional Medicare beneficiaries, we identified the first telemental health visit (hereinafter, *index visit*) between patient and clinician (using the National Provider Identifier; eAppendix in Supplement 1). We excluded index visits for patients not enrolled in Medicare in the prior 12 months and for those with substance use disorders.

For 2019-2022 index visits, we captured whether there was an in-person visit for any reason with the same clinician in the prior 3, 6, or 12 months. The forthcoming rule requires an in-person visit within 6 months; because of policy debate about the most appropriate timing, we also explored other periods. In other contexts, Medicare allows a visit with a clinician from the same specialty within the same practice.^5^ We explored this Medicare flexibility in telemental health and measured how often a patient had a prior in-person visit with a clinician from the same specialty (or with any clinician) in the same practice (via Taxpayer Identification Number).

For 2022 index visits, we compared patients with and without an in-person visit in the prior 6 months on characteristics including age, clinician specialty, and clinician-patient distance (eAppendix in Supplement 1). We conducted sensitivity analyses excluding rural residents (who will not have in-person visit requirements); results were similar. Data were analyzed in November 2023 using SAS Enterprise Guide, version 7.15 (SAS Institute).

## Results

There were 4 162 441 index visits between 2019 and 2022. Visits peaked in April 2020 and then decreased (Figure A). In 2022, there were 205 463 index visits across 197 430 patients.

**Figure.**
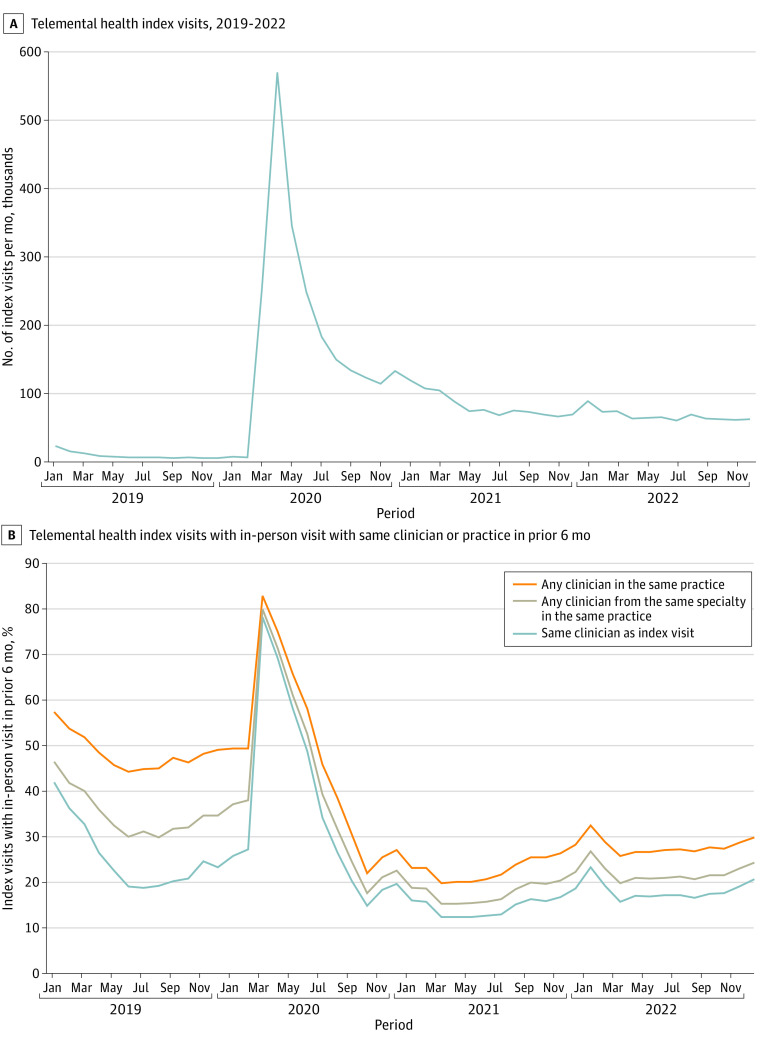
Index Telemental Health Visits Between Patient and Clinician Specialty had to be the same as the specialty of clinician on the index visit. For example, if the index telemental health visit was with a social worker, then we noted whether there were any social worker visits in the prior 6 months at the same practice. Specialties are as categorized by Medicare (eAppendix in Supplement 1).

Across 2019-2022 index visits, 44.2% of patients had any prior in-person visit with the same clinician. Among index visits with a prior in-person visit, the most proximal in-person visit occurred a median 86 days prior (IQR, 41-182). Of all 2019-2022 index visits, 26.8%, 34.5%, and 39.4% of patients had an in-person visit with the same clinician in the prior 3, 6, and 12 months, respectively. These rates varied over time (Figure B). In 2022, 18.4%, 28.2%, and 22.3% of patients with index visits had an in-person visit with the same clinician, any clinician in the same practice, or a clinician from the same specialty in the same practice in the prior 6 months, respectively.

As clinician-patient distance increased, the proportion of 2022 index visits with an in-person visit in the prior 6 months decreased (Table). Black and Hispanic patients were less likely to have an in-person visit in the prior 6 months.

**Table.** Index Telemental Health Visits With an In-Person Visit in the Prior 6 Months, 2022

Characteristic	Index telemental health visits		*P* value
	All (N = 814 303)	With an in-person visit in prior 6 mo (n = 149 956 [18.4%])^a^	
Age group, y			
<65	313 670	58 254 (18.6)	<.001
65-74	312 720	58 743 (18.8)	
75-84	139 773	26 168 (18.7)	
≥85	48 140	6791 (14.1)	
Sex^b^			
Male	272 361	49 474 (18.2)	<.001
Female	541 465	100 420 (18.5)	
Race and ethnicity^c^			
Black	74 107	11 565 (15.6)	<.001
Hispanic	54 729	8673 (15.8)	
White	633 386	120 769 (19.1)	
Other^d^	25 232	4198 (16.6)	
Unknown	26 849	4751 (17.7)	
Dually eligible for Medicaid			
No	457 158	88 793 (19.4)	<.001
Yes	357 145	61 163 (17.1)	
Patient lives in rural community^e^			
No	662 190	119 812 (18.1)	<.001
Yes	152 113	30 144 (19.8)	
Index visit for serious mental illness			
No	674 725	121 525 (18.0)	<.001
Yes	139 578	28 431 (20.4)	
Specialty of clinician on index visit^f^			
Primary care	265 219	42 517 (16.0)	<.001
Mental health specialist	491 831	97 734 (19.9)	
Other	57 253	9705 (17.0)	
Distance between clinician and patient, miles^g^			
0-1	81 118	19 540 (24.1)	<.001
2-5	132 889	27 552 (20.7)	
6-10	137 013	28 667 (20.9)	
11-30	235 856	44 223 (18.8)	
31-60	93 300	14 785 (15.8)	
≥60	133 475	15 093 (11.3)	

^a^ Values are listed as No. (% of total) of in-person visits in the prior 6 months with the same clinician who provided the first telemental health visit. Row percentages (ie, of the index visits in a given row, the proportion that had an in-person visit within the prior 6 months) are presented. A χ^2^ test was used to compare receipt of an in-person visit for each demographic variable.

^b^ Missing for 477 index telemental health visits.

^c^ The Medicare Research Triangle Institute variable combines race and ethnicity in a single variable.

^d^ Includes American Indian or Alaska Native, Asian or Pacific Islander, and other race or ethnicity (eAppendix in Supplement 1).

^e^ Captured based on Medicare designation of communities as rural for telemedicine reimbursement.

^f^ Captured by Medicare specialty code on index visit. Mental health specialists included social workers, psychiatrists, psychologists, and psychiatric mental health nurse practitioners (detailed in the eAppendix in Supplement 1).

^g^ Distance between the centroids of the zip codes of the patient’s address and the clinician’s address. One zip code was missing for 652 index telemental health visits. To convert miles to kilometers, multiply by 1.6.

## Discussion

Congress mandated that starting in 2025, Medicare patients initiating telemental health care must have an in-person visit in the prior 6 months. Here, approximately 1 in 5 index visits in 2022 were preceded by an in-person visit. A key limitation of our distance measure is that we did not differentiate between multiple physical sites in the same practice.

These findings suggest that Medicare’s in-person visit requirement will require substantial changes in current practice. Future work should assess quality benefits of requiring in-person care. Medicare could consider increasing flexibility for in-person visit requirements to minimize disruptions in care.

## Notes

Supplement 1.
eAppendix

Supplement 2.
Data Sharing Statement
