# Supplementary material for: In-Person Visits Before Initiation of Telemedicine for Mental Illness
Source: JAMA Health Forum. 2024 Apr 5;5(4):e240234. doi: 10.1001/jamahealthforum.2024.0234 (PMC10998149; doi:10.1001/jamahealthforum.2024.0234)
Supplement: Supplement 1. — eAppendix [file jamahealthforum-e240234-s001.pdf]

Supplementary Online Content

Mehrotra A, Busch AB, Uscher-Pines L, Raja P, Huskamp HA. In-person visits before initiation of telemedicine for mental illness. *JAMA Health Forum*. 2024;5(3):e240234. doi:10.1001/jamahealthforum.2024.0234

eAppendix

This supplementary material has been provided by the authors to give readers additional information about their work.

© 2024 Mehrotra A et al. *JAMA Health Forum*.

eAppendix

Definition of mental health visits.

Building off prior work, we used both HCPCS/CPT codes and diagnosis codes to identify mental health visits.

- The full list of HCPCS/CPT codes to define visits are available in prior work (Mehrotra A, Huskamp HA, Souza J, Uscher-Pines L, Rose S, Landon BE, et al. Rapid growth in mental health telemedicine use among rural Medicare beneficiaries, wide variation across states. Health Aff (Millwood). 2017;36(5))
- A mental health visit was a visit in which the primary (first) diagnosis code for the visit was ICD-10: F20-F69, F80–F99. We excluded visits with a substance use disorder diagnosis because these visits will not be subject to the in-person requirement when it goes into effect.

Definition of telemedicine visits

Telemedicine were visits with modifier codes of (GT, GQ, FQ, 95, 93) or a place of service code (02,10) or telemedicine-specific visit codes: G0406-8, 99441–3, 98966–8.

In recent rulemaking, CMS has indicated that clinicians should flag telemedicine visits focused on telemental health using FQ and FR modifier codes. However, these were not used during the entire study period.

Clinical specialty

A mental health specialist was defined as a psychologist, psychiatrist, social worker, or psychiatric mental health nurse practitioner (PMHNP). All were defined using Medicare's specialty codes (https://resdac.org/cms-data/variables/line-cms-provider-specialty-code) except PMHNPs. There is no designated specialty code for nurse practitioners that specialize in mental health treatment. To identify these nurse practitioners, we use an algorithm previously published that categorizes nurse practitioners based on the conditions they treat (Richard, Jessica V., et al. "A methodology for identifying behavioral health advanced practice registered nurses in administrative claims." Health Services Research 57.4 (2022): 973-978.).

Primary care was defined as internal medicine, family medicine, and geriatrics.

Demographic characteristics and other characteristics of index telemental health visits

For the purposes of Medicare reimbursement for telemedicine, Medicare has designated rurality as being outside of those who live outside a Metropolitan Core Based Statistical Area or those within an area assigned a rural urban commuting area code 4–10.

Medicare has two variables available to capture race and ethnicity. We used the RTI variable (https://resdac.org/cms-data/variables/research-triangle-institute-rti-race-code) as it has higher sensitivity for identifying Hispanic individuals. Given prior evidence that Asian/Pacific Islander and American Indian / Alaska Native designation has low sensitivity, we included these groups

© 2024 Mehrotra A et al. *JAMA Health Forum*.

into the Other category along with those designated as “Other” by the variable. White designated Non-Hispanic White.

Serious mental illness was defined as those index telemental health visits where the first diagnosis schizophrenia (F20-F29) or bipolar-I disorder (F30, F31.0-31.7, F31.9).

Distance captured the distance in miles between the centroids of the patient’s home zip code and the zip code for the clinician’s practice. There are several limitations to this measure. The patient’s zip code of residence may be inaccurate because, for example, the patient travels to a clinic from their work, the patient may have multiple residences, or the address in the Medicare beneficiary file may be for a family member (e.g., patient lives in a nursing home). A clinician may have multiple practices and the zip code in the data might not capture the closest practice. Finally, we acknowledge that distance does not always correlate with travel time. Travel times vary by time of day, are slower in urban areas due to traffic and many patients take public transportation. This is particularly important for lower-income patients who may not have their own vehicle.

Analyses were conducted in SAS Enterprise Guide version 7.15 (Cary, NC).

© 2024 Mehrotra A et al. *JAMA Health Forum*.
