# Supplementary material for: In-Person Visits Before Initiation of Telemedicine for Mental Illness
Source: JAMA Health Forum. 2024 Apr 5;5(4):e240234. doi: 10.1001/jamahealthforum.2024.0234 (PMC10998149; doi:10.1001/jamahealthforum.2024.0234)
Supplement: Supplement 2. — Data Sharing Statement [file jamahealthforum-e240234-s002.pdf]

Data Sharing Statement

Mehrotra. In-Person Visits Before Initiation of Telemedicine for Mental Illness. *JAMA Health Forum*. Published April 05, 2024. doi:10.1001/jamahealthforum.2024.0234

Data
Data available: No

Additional Information
Explanation for why data not available: The data and code used in this analysis are protected by a data use agreement with the Centers for Medicare and Medicaid Services. The authors can be contacted for more details about our analyses.
